# Evaluating equivalent square field size definitions for rectangular small fields

**DOI:** 10.1002/acm2.70500

**Published:** 2026-02-10

**Authors:** Ignasi Méndez, Mateb Al Khalifa, Haya Aljuaid, Božidar Casar

**Affiliations:** ^1^ Department of Dosimetry and Quality of Radiological Procedures Institute of Oncology Ljubljana Ljubljana Slovenia; ^2^ Virginia Commonwealth University Richmond Virginia USA; ^3^ Department of Radiation Medicine Northwell Health Lake Success New York USA

**Keywords:** equivalent square field size, field output factors, radiochromic films, small fields dosimetry

## Abstract

**Background:**

In the IAEA TRS‐483 Code of Practice (CoP), rectangular small field sizes are approximated to equivalent square small fields using the definition proposed by Cranmer‐Sargison et al. However, the CoP estimates the uncertainties associated with this formula only for rectangular fields with dimensions within the range 0.7<X/Y<1.4.

**Purpose:**

The objective of the present study was to compare the accuracy of the Cranmer‐Sargison definition with other formulas for equivalent square small fields in the context of measuring field output factors (FOFs) for rectangular small fields, both within and outside the range covered by the CoP.

**Methods:**

Measurements were conducted using Gafchromic EBT4 radiochromic films. The models compared included Cranmer‐Sargison, Sterling, Superellipse, Sterling‐Partial Superellipse, Sterling‐Superellipse, Vadash and Bjärngard, and Fogliata. The most accurate definition of equivalent square field size was identified as the one yielding the lowest discrepancy between measured and analytical values, with the log‐likelihood of the measurements selected as the metric. Analytical values were derived using the function introduced by Sauer and Wilbert, which relates FOFs to equivalent square field sizes.

**Results:**

The best results were achieved with the Fogliata model, followed in terms of accuracy by the Sterling‐Partial Superellipse model. The Sterling‐Superellipse and Vadash and Bjärngard models came next. It should be noted that the Sterling‐Partial Superellipse and Sterling‐Superellipse models rely solely on the geometric shape of the irradiation field size, whereas the Fogliata and Vadash and Bjärngard models incorporate a fitting parameter. The Sterling definition, while less accurate than these models, improved upon the Cranmer‐Sargison definition and retained computational simplicity. Finally, the Cranmer‐Sargison and Superellipse models exhibited the largest discrepancies.

**Conclusions:**

This study identified several definitions of equivalent square small field size that could refine the IAEA TRS‐483 CoP by improving the accuracy of field output correction factors for rectangular small fields. Among these definitions, the Fogliata model obtained the best results.

## 1 INTRODUCTION

The IAEA TRS‐483 Code of Practice (CoP), jointly developed by the IAEA and the AAPM, addresses the dosimetry of small static fields used in external beam radiotherapy.^1^ Among the topics covered by this international CoP are field output correction factors, for which tabulated values are provided as a function of small square field size. Nonsquare small fields are approximated to have the same output correction factors as square fields with equal area.^1^, ^2^ This definition of equivalent square small field size was proposed by Cranmer‐Sargison et al.^3^, and for rectangular fields, it can be expressed as follows:

(1) $$S_{clin}=\sqrt{XY}$$

where X and Y represent cross‐plane and in‐plane dimensions, respectively, and field size dimensions are defined by the full width at half maximum (FWHM) of the lateral profile. The CoP estimates the uncertainties derived from using this formula for rectangular fields with dimensions within 0.7<X/Y<1.4. Outside these limits, uncertainties are expected to increase.

A commonly used alternative definition of equivalent square fields, which has been shown to produce a better approximation for small rectangular fields,^4^, ^5^ is the Sterling area‐to‐perimeter formula^6^:

(2) $$S_{sterling}=\frac{4\;\mathrm{Area}}{\mathrm{Perimeter}}=\frac{2\;XY}{\left(X+Y\right)}$$

In a previous study, equivalent square small fields were defined based on the concept of the superellipse.^7^ Superellipses are a family of curves that encompass ellipses and rectangles. Irradiation fields of square, rectangular, and circular fields are better and uniformly characterized by superellipses, following the formula:

(3) $${\left|\frac{x}{X/2}\right|}^{n}+{\left|\frac{y}{Y/2}\right|}^{n}=1$$

where, along with the cross‐plane (X) and in‐plane (Y) dimensions, a third parameter is used, the degree (n), which is also determined from the shape of the irradiation field, described by the coordinates (x, y).

To calculate the area of the superellipse (${\mathrm{Area}}_{se}$), a correction factor involving the gamma function and the degree n is applied to the area of a rectangle

(4) $${\mathrm{Area}}_{se}=XY\frac{{\left(\Gamma \left(1+\frac{1}{n}\right)\right)}^{2}}{\Gamma \left(1+\frac{2}{n}\right)}$$

Thus, an alternative definition for equivalent square small fields based on the concept of the superellipse was formulated as follows:

(5) $$S_{se}=\sqrt{XY}\frac{\Gamma \left(1+\frac{1}{n}\right)}{\sqrt{\Gamma \left(1+\frac{2}{n}\right)}}$$

In the study presented here, two new alternative definitions, which combine the superellipse and Sterling formulas, are introduced:

- Sterling‐Partial Superellipse:
(6) $$S_{sp}=\frac{4\;\mathrm{Area}}{{\mathrm{Perimeter}}_{se}}$$
- Sterling‐Superellipse:
(7) $$S_{ss}=\frac{4\;{\mathrm{Area}}_{se}}{{\mathrm{Perimeter}}_{se}}$$

Since the perimeter of a superellipse (${\mathrm{Perimeter}}_{se}$), like that of an ellipse, cannot be expressed analytically, the approximation published by Erbaş^8^ was used:

(8) $${\mathrm{Perimeter}}_{se}=\max\left(\frac{X}{2},\frac{Y}{2}\right)P\left(h\right)$$

where $h=\min\left(\frac{X}{Y},\frac{Y}{X}\right)$ and the term *P*(*h*) is defined as follows:

(9) $$\begin{array}{cc} P(h) & =\left(1.176{\left(1-\frac{1}{n}\right)}^{1.89}+2.825\right)\left(1+h\right)\\ & +\frac{\left(-0.03n^{2}+25n-16.6\right){\left(1-h\right)}^{2}}{\left(10n^{2}+0.2n-7.6\right)\left(1+h\right)+\left(n^{2}-1.5n+5\right)\sqrt{1+240{\left(1-\frac{1}{n}\right)}^{4.74}h+h^{2}}} \end{array}$$

None of the previous definitions accounts for the collimator‐exchange effect. This effect refers to output differences for a rectangular field, depending on whether the long side is formed by the upper or lower jaw.^9^ Vadash and Bjärngard^10^ introduced an empirical parameter (A) in their definition of equivalent square fields to correct for this:

(10) $$S_{vb}=\frac{(A+1)XY}{\left(AX+Y\right)}$$

Recently, Fogliata et al.^11^ introduced a novel formula that also includes a fitting parameter (a) to account for the collimator‐exchange effect:

(11) $$S_{fo}=\frac{2\min{\left(X,Y\right)}^{a}\max{\left(X,Y\right)}^{\left(2-a\right)}}{\left(X+Y\right)}$$

This expression can be rewritten in the form proposed by Bjärngard and Siddon,^12^ as the Sterling formula with a correction term^11^:

(12) $$S_{fo}=\frac{2\;XY}{\left(X+Y\right)}{\left(\frac{\min\left(X,Y\right)}{\max\left(X,Y\right)}\right)}^{a-1}$$

The purpose of the present work was to compare the accuracy of equation (1) against other definitions of equivalent square small fields, both within and beyond the range covered by the CoP. Cranmer‐Sargison formula was compared to Sterling, Superellipse, Sterling‐Partial Superellipse, Sterling‐Superellipse, Vadash and Bjärngard, and Fogliata definitions of equivalent square small fields, which correspond to Equations (1), (2), (5), (6), (7), (10), and (11), respectively. Among this set of definitions, only the Vadash and Bjärngard and Fogliata formulas include a fitting parameter. To prevent loss of predictive accuracy due to overfitting, formulas with more parameters, such as Thomas et al.^13^ or Zhou et al.^14^ models, were discarded.

## 2 METHODS AND MATERIALS

### 2.1 Measurements with radiochromic films

#### 2.1.1 Film irradiation

To evaluate the accuracy of each model, the field output factors (FOFs) for 39 different nominal field sizes were measured: 10 cm × 10 cm, 5 cm × 5 cm, 4 cm × 4 cm, and all combinations where the *X* and *Y* dimensions were 3, 2, 1.5, 1, 0.8, or 0.5 cm (e.g., 3 cm × 3 cm, 3 cm × 2 cm, 2 cm × 3 cm, etc.). Each FOF was measured three times with radiochromic films Gafchromic EBT4 (Ashland Inc., Wayne, NJ, USA) from the same lot. All films were irradiated on a Varian TrueBeam linear accelerator (Varian Medical Systems, Palo Alto, CA, USA) with 6‐MV energy and flattening filter (WFF). The films were placed in a Solid Water HE phantom (Sun Nuclear, Melbourne, FL, USA) at a depth of 10 cm, with source‐to‐axis distance (SAD) of 100 cm, and were exposed to 500 monitor units (MU). To convert film scan pixel values into absorbed doses, a calibration was performed. For this purpose, a set of seven film strips with dimensions 20.3 cm × 3.5 cm was irradiated using the same setup as the FOFs, but with a fixed nominal field size of 25 cm × 25 cm and 0, 50, 100, 200, 300, 400, and 500 MU, respectively. All field sizes were collimated using only the jaws. There is a constant factor between the doses for 25 cm × 25 cm and 10 cm × 10 cm field sizes. However, multiplying the calibration doses by a constant factor does not affect the calculation of FOFs. The 25 cm × 25 cm field size was chosen to include the calculation of the lateral correction^15^, ^16^, ^17^, ^18^ in the calibration. As part of this procedure, an unexposed whole film was scanned.^19^

FOF and calibration films were scanned before and after irradiation to improve measurement accuracy by mitigating film heterogeneity.^19^, ^20^ A PMMA frame was used to ensure consistent placement of the films. Films were scanned in transmission mode at a resolution of 50 dpi for calibration and 150 dpi for FOF films. Four repeated scans for each film were taken, the first image was discarded, and the mean image of the remaining three scans was calculated. Scans were performed on an Epson Expression 12000XL (Seiko Epson Corporation, Nagano, Japan) with scan software Epson Scan 2. Color corrections were disabled during scanning and all images were saved as 48‐bit RGB TIFF files.

#### 2.1.2 Film analysis

Film scans were processed and analyzed using Radiochromic.com v5.1 (Radiochromic SL, Benifaió, Spain). The lateral correction of the film‐scanner system was determined using the calibration films together with the unexposed film^19^ and applied to the FOF films. Film pixel values were converted to doses using the Multigaussian model for multichannel film dosimetry^20^ implemented in Radiochromic.com. The software also includes a dedicated Radiation Field functionality for FOF measurements, which calculates field dimensions based on the FWHM, the degree (n) of the superellipse describing the field, statistics of the absorbed dose within a region of interest (ROI) centered on the field, and the volume‐averaging correction ($k_{vol}$) for the central ROI following Casar et al. ^21^ For the central ROI, a diameter of 0.5 mm was chosen.^21^, ^22^

Thus, from each FOF film, the field dimensions, degree n of the superellipse, $k_{vol}$, and absorbed dose statistics (mean dose, standard deviation, and number of measuring points) were obtained. The expected values and uncertainties of the field dimensions, degree n, and corrected central dose (mean central dose multiplied by $k_{vol}$) were calculated by combining the three FOF measurements for each nominal field size. Expected values were taken as the mean of the measurements.

The uncertainties of the film doses accounted for intrafilm, interfilm, and intralot variations. Uncertainties of the field dimensions were obtained by combining statistical (Type A) uncertainties with conservative Type B estimates based on film dose uncertainties and the resolution of the FOF scans, resulting in a Type B uncertainty of 0.01 cm for each dimension.^23^ For the degree n, uncertainties combined Type A uncertainties with Type B uncertainties obtained via parametric bootstrap resampling of the points defining the FWHM isodose. The relative Type B uncertainty of n was 2%. In this study, all uncertainties are expressed with a coverage factor of k=1.

### 2.2 Equivalent square small field sizes

With mean values and uncertainties of the field dimensions (X and Y) and the degree (n) of the superellipse, equivalent square small field sizes for all nominal field sizes across all models under comparison were computed. The uncertainties were calculated with error propagation with the exception of the Superellipse, Sterling‐Partial Superellipse, and Sterling‐Superellipse models, which were calculated with parametric bootstrap resampling.

### 2.3 Field output factors

Following the formalism of Alfonso et al.,^24^ FOFs are calculated as follows:

(13) $$\Omega_{Q_{clin},Q_{ref}}^{f_{clin},f_{ref}}=\frac{D_{w,Q_{clin}}^{f_{clin}}}{D_{w,Q_{ref}}^{f_{ref}}}=\frac{M_{Q_{clin}}^{f_{clin}}}{M_{Q_{ref}}^{f_{ref}}}k_{Q_{clin},Q_{ref}}^{f_{clin},f_{ref}}$$

In this equation, $Q_{clin}$ and $Q_{ref}$ are the beam qualities of $f_{clin}$ and $f_{ref}$, which denote clinical and reference fields, respectively. The calculation as ratio of doses (D) is equivalent to the ratio of detector responses (M) multiplied by a correction factor $k_{Q_{clin},Q_{ref}}^{f_{clin},f_{ref}}$.

In this work, the reference field $f_{ref}$ was S=10cm, where S symbolizes the equivalent square small field size, and the correction factor for film measurements was $k_{vol}$. Thus,

(14) $$\Omega {\left(S\right)}^{exp}=\Omega_{Q_{clin},Q_{ref}}^{f_{clin},f_{ref}}=\frac{M(S)}{M(S=10\;\mathrm{cm})}k_{vol}\left(S\right)$$

$\Omega_{Q_{clin},Q_{ref}}^{f_{clin},f_{ref}}$ is represented as $\Omega {\left(S\right)}^{exp}$, which will denote the analytical function introduced by Sauer and Wilbert^25^ of the FOF as a function of equivalent square field size:

(15) $$\Omega {\left(S\right)}^{fit}=P_{\infty }\frac{S^{m}}{l^{m}+S^{m}}+S_{\infty }\left(1-e^{-bS}\right),$$

where $P_{\infty }$, $S_{\infty }$, m, l, and b are fitting parameters.

Using only nominal square fields, $\Omega {\left(S\right)}^{fit}$ was fitted, which in turn yielded M(S=10cm), for each definition of the equivalent square field size (S). Mean values and uncertainties of corrected central doses and equivalent square small field sizes were included in the fit. The uncertainties of $\Omega {\left(S\right)}^{fit}$ were derived with parametric bootstrap resampling, while the uncertainties of $\Omega {\left(S\right)}^{exp}$ were obtained through error propagation.

### 2.4 Model comparison

To determine the accuracy of each definition of equivalent square field size, the distance between measured ($\Omega {\left(S\right)}^{exp}$) and analytical ($\Omega {\left(S\right)}^{fit}$) FOFs was evaluated for all the fields. To account for not only the mean values but also the uncertainties of the FOFs, the relative log‐likelihood was used as metric:

(16) $$L\left(M\right)=-\sum_{i}\left(\log\left(\sigma_{i}\right)+{\left(\frac{\Delta_{i}}{2\sigma_{i}}\right)}^{2}\right),$$

where L is the log‐likelihood, M is a model of equivalent square small field size, i is a particular field, Δ is the distance between measured and analytical FOFs, and σ is the uncertainty of Δ, calculated as the combined uncertainty of $\Omega {\left(S\right)}^{exp}$ and $\Omega {\left(S\right)}^{fit}$.

For the Vadash and Bjärngard and Fogliata formulas, the empirical parameters A and a were optimized to maximize their log‐likelihood.

Therefore, it was considered that the most accurate definitition of equivalent square field size yielded the lowest discrepancy between measured and analytical values for rectangular fields, and consequently had the largest value of L.

## 3 RESULTS

### 3.1 Equivalent square small field sizes and FOFs

Table 1 presents field dimensions (*X* and *Y*), degree (n) of the superellipse, and equivalent square small field sizes calculated according to the definitions under comparison ($S_{clin}$, $S_{sterling}$, $S_{se}$, $S_{sp}$, $S_{ss}$, $S_{vb}$, and $S_{fo}$) for all nominal field sizes. The fitting parameters that maximized L for $S_{vb}$ and $S_{fo}$ were A=1.5 and a=1.12, respectively.

**TABLE 1:** For all nominal field sizes, field dimensions (*X* and *Y*), degree (n) of the superellipse, and equivalent square small field sizes calculated according to the definitions under comparison ($S_{clin}$, $S_{sterling}$, $S_{se}$, $S_{sp}$, $S_{ss}$, $S_{vb}$, and $S_{fo}$).

Nom. field	*X*	*Y*	*n*	$S_{clin}$	$S_{sterling}$	$S_{se}$	$S_{sp}$	$S_{ss}$	$S_{vb}$	$S_{fo}$
(cm × cm)	(cm)	(cm)		(cm)	(cm)	(cm)	(cm)	(cm)	(cm)	(cm)
0.5 × 0.5	0.49	0.53	2.44(8)	0.508(7)	0.507(7)	0.465(7)	0.622(9)	0.522(8)	0.511(7)	0.503(8)
0.5 × 0.8	0.50	0.76	2.84(9)	0.615(8)	0.601(9)	0.574(7)	0.714(12)	0.623(10)	0.627(8)	0.571(9)
0.5 × 1.0	0.50	0.95	3.09(7)	0.692(8)	0.658(9)	0.652(7)	0.767(11)	0.681(10)	0.701(9)	0.610(10)
0.5 × 1.5	0.51	1.42	3.62(14)	0.849(9)	0.748(11)	0.812(9)	0.839(14)	0.767(12)	0.826(11)	0.660(11)
0.5 × 2.0	0.51	1.93	4.13(22)	0.996(11)	0.812(14)	0.961(11)	0.889(18)	0.828(15)	0.919(14)	0.693(14)
0.5 × 3.0	0.51	2.91	5.41(30)	1.222(14)	0.873(17)	1.196(13)	0.925(19)	0.885(19)	1.015(18)	0.709(15)
0.8 × 0.5	0.76	0.54	2.96(9)	0.636(7)	0.627(8)	0.597(8)	0.742(10)	0.654(9)	0.606(8)	0.601(8)
0.8 × 0.8	0.77	0.77	3.31(7)	0.769(7)	0.769(7)	0.730(7)	0.899(9)	0.810(8)	0.770(7)	0.768(8)
0.8 × 1.0	0.77	0.95	3.62(10)	0.856(7)	0.851(8)	0.818(8)	0.981(9)	0.897(8)	0.870(7)	0.829(8)
0.8 × 1.5	0.78	1.44	4.04(15)	1.062(9)	1.014(10)	1.023(9)	1.144(13)	1.062(13)	1.078(10)	0.943(11)
0.8 × 2.0	0.78	1.93	4.79(15)	1.229(9)	1.113(10)	1.196(9)	1.223(12)	1.157(11)	1.216(10)	0.998(11)
0.8 × 3.0	0.78	2.91	6.19(15)	1.510(10)	1.235(13)	1.484(9)	1.315(14)	1.270(14)	1.395(13)	1.055(12)
1.0 × 0.5	0.94	0.54	3.36(10)	0.712(8)	0.685(9)	0.677(7)	0.791(11)	0.714(9)	0.649(9)	0.640(9)
1.0 × 0.8	0.96	0.78	3.63(9)	0.863(7)	0.859(7)	0.825(7)	0.990(9)	0.905(8)	0.842(8)	0.838(8)
1.0 × 1.0	0.96	0.96	4.00(9)	0.963(7)	0.963(7)	0.927(7)	1.098(8)	1.018(8)	0.963(7)	0.963(7)
1.0 × 1.5	0.97	1.44	4.66(11)	1.180(8)	1.158(8)	1.146(8)	1.293(10)	1.220(9)	1.205(8)	1.104(9)
1.0 × 2.0	0.97	1.94	5.33(22)	1.375(9)	1.298(10)	1.344(9)	1.421(12)	1.358(12)	1.390(9)	1.195(11)
1.0 × 3.0	0.98	2.92	6.50(18)	1.691(9)	1.467(12)	1.664(10)	1.567(14)	1.518(13)	1.629(12)	1.286(12)
1.5 × 0.5	1.45	0.55	3.73(13)	0.897(9)	0.801(11)	0.859(9)	0.899(13)	0.825(13)	0.735(11)	0.713(11)
1.5 × 0.8	1.46	0.79	4.32(13)	1.075(8)	1.026(9)	1.040(8)	1.149(12)	1.076(11)	0.969(10)	0.954(10)
1.5 × 1.0	1.46	0.97	4.68(11)	1.192(8)	1.168(8)	1.158(7)	1.303(9)	1.230(9)	1.123(9)	1.112(9)
1.5 × 1.5	1.48	1.45	5.41(12)	1.464(7)	1.464(7)	1.432(7)	1.615(9)	1.545(8)	1.461(7)	1.461(7)
1.5 × 2.0	1.47	1.94	6.29(16)	1.691(7)	1.675(8)	1.663(7)	1.822(9)	1.761(9)	1.723(7)	1.620(8)
1.5 × 3.0	1.48	2.93	7.70(20)	2.081(8)	1.965(9)	2.057(8)	2.095(11)	2.047(10)	2.103(9)	1.810(10)
2.0 × 0.5	1.96	0.55	4.36(21)	1.043(10)	0.865(13)	1.009(9)	0.945(15)	0.885(14)	0.778(12)	0.743(15)
2.0 × 0.8	1.96	0.79	5.01(29)	1.246(9)	1.128(11)	1.215(8)	1.235(14)	1.173(13)	1.040(11)	1.012(11)
2.0 × 1.0	1.96	0.97	5.41(13)	1.383(8)	1.302(9)	1.352(8)	1.424(12)	1.362(10)	1.220(10)	1.197(10)
2.0 × 1.5	1.97	1.45	6.36(14)	1.692(7)	1.672(8)	1.664(8)	1.817(9)	1.757(8)	1.623(8)	1.612(8)
2.0 × 2.0	1.97	1.95	7.30(16)	1.962(7)	1.962(7)	1.937(7)	2.112(8)	2.058(7)	1.959(7)	1.959(7)
2.0 × 3.0	1.98	2.94	8.84(18)	2.413(8)	2.368(8)	2.392(8)	2.513(9)	2.468(9)	2.463(8)	2.259(9)
3.0 × 0.5	2.95	0.56	5.73(25)	1.281(12)	0.936(15)	1.256(13)	0.991(17)	0.952(15)	0.824(14)	0.766(13)
3.0 × 0.8	2.96	0.80	6.18(21)	1.533(10)	1.253(13)	1.507(10)	1.335(14)	1.289(13)	1.124(12)	1.070(12)
3.0 × 1.0	2.97	0.98	6.49(14)	1.703(9)	1.471(12)	1.676(9)	1.571(13)	1.522(13)	1.336(11)	1.288(12)
3.0 × 1.5	2.97	1.46	7.67(17)	2.082(8)	1.956(9)	2.057(7)	2.086(11)	2.037(10)	1.831(10)	1.796(10)
3.0 × 2.0	2.98	1.96	8.70(20)	2.414(7)	2.361(8)	2.391(7)	2.508(9)	2.462(9)	2.268(8)	2.245(9)
3.0 × 3.0	2.98	2.95	10.77(22)	2.966(7)	2.966(7)	2.947(7)	3.119(8)	3.080(8)	2.962(8)	2.962(7)
4.0 × 4.0	4.01	3.97	13.36(29)	3.987(7)	3.987(7)	3.971(7)	4.153(9)	4.119(8)	3.983(7)	3.982(7)
5.0 × 5.0	5.02	4.97	16.84(35)	4.993(7)	4.993(7)	4.980(8)	5.158(8)	5.130(8)	4.988(7)	4.987(7)
10.0 × 10.0	10.07	9.97	29.56(60)	10.021(7)	10.021(7)	10.012(8)	10.208(8)	10.189(8)	10.012(7)	10.010(7)

*Note*: The uncertainties (*k* = 1) are expressed in concise notation. The uncertainties of *X* and *Y* were estimated as 0.01 cm.

Table 2 shows analytical FOFs for all nominal field sizes and all definitions of equivalent field size under comparison. Additionally, $\Omega {\left(S_{clin}\right)}^{exp}$ measures are also included.

**TABLE 2:** For all nominal field sizes, measured field output factors normalized with $M(S_{clin}=10\;\mathrm{cm})$, as well as field output factors fitted with the analytical function proposed by Sauer and Wilbert for each definition of equivalent square small field size under comparison.

Nom. field ($cm^{2}$)	$\Omega {\left(S_{clin}\right)}^{exp}$	$\Omega {\left(S_{clin}\right)}^{fit}$	$\Omega {\left(S_{sterling}\right)}^{fit}$	$\Omega {\left(S_{se}\right)}^{fit}$	$\Omega {\left(S_{sp}\right)}^{fit}$	$\Omega {\left(S_{ss}\right)}^{fit}$	$\Omega {\left(S_{vb}\right)}^{fit}$	$\Omega {\left(S_{fo}\right)}^{fit}$
0.5 × 0.5	0.463(9)	0.462(12)	0.462(10)	0.462(11)	0.464(11)	0.463(12)	0.462(10)	0.463(11)
0.5 × 0.8	0.552(5)	0.541(8)	0.533(8)	0.542(7)	0.528(8)	0.532(8)	0.547(7)	0.514(8)
0.5 × 1.0	0.572(4)	0.584(8)	0.568(8)	0.586(7)	0.559(8)	0.565(8)	0.589(8)	0.540(8)
0.5 × 1.5	0.601(6)	0.649(8)	0.612(9)	0.650(8)	0.596(8)	0.605(8)	0.642(8)	0.569(7)
0.5 × 2.0	0.608(7)	0.691(8)	0.638(10)	0.691(8)	0.618(9)	0.629(8)	0.671(8)	0.586(8)
0.5 × 3.0	0.609(9)	0.733(7)	0.659(10)	0.734(7)	0.632(9)	0.648(8)	0.696(8)	0.594(8)
0.8 × 0.5	0.516(10)	0.554(8)	0.549(8)	0.556(7)	0.545(8)	0.550(8)	0.534(7)	0.534(8)
0.8 × 0.8	0.622(6)	0.620(8)	0.621(9)	0.621(8)	0.622(9)	0.622(8)	0.620(8)	0.619(8)
0.8 × 1.0	0.653(12)	0.651(8)	0.651(10)	0.652(8)	0.651(9)	0.651(8)	0.657(8)	0.642(8)
0.8 × 1.5	0.687(14)	0.705(8)	0.696(9)	0.705(8)	0.695(9)	0.694(8)	0.709(8)	0.677(8)
0.8 × 2.0	0.700(6)	0.734(7)	0.716(9)	0.734(7)	0.711(8)	0.713(8)	0.733(7)	0.690(8)
0.8 × 3.0	0.713(7)	0.767(6)	0.736(8)	0.768(7)	0.728(8)	0.731(7)	0.756(7)	0.703(8)
1.0 × 0.5	0.532(11)	0.594(8)	0.582(9)	0.598(7)	0.572(8)	0.581(8)	0.560(7)	0.558(7)
1.0 × 0.8	0.642(15)	0.654(8)	0.654(10)	0.655(8)	0.654(9)	0.654(8)	0.647(8)	0.645(8)
1.0 × 1.0	0.679(17)	0.682(8)	0.684(9)	0.683(8)	0.684(9)	0.684(8)	0.683(8)	0.682(8)
1.0 × 1.5	0.728(9)	0.726(7)	0.724(8)	0.727(7)	0.724(8)	0.723(7)	0.731(8)	0.712(8)
1.0 × 2.0	0.738(5)	0.753(6)	0.744(8)	0.753(7)	0.743(7)	0.743(7)	0.756(7)	0.728(7)
1.0 × 3.0	0.753(4)	0.783(6)	0.763(7)	0.783(6)	0.760(7)	0.761(7)	0.779(7)	0.741(7)
1.5 × 0.5	0.547(12)	0.664(8)	0.634(9)	0.665(8)	0.621(9)	0.628(8)	0.605(8)	0.595(8)
1.5 × 0.8	0.673(12)	0.707(8)	0.699(9)	0.708(8)	0.696(8)	0.697(8)	0.685(8)	0.679(8)
1.5 × 1.0	0.716(14)	0.728(7)	0.725(8)	0.729(7)	0.726(8)	0.725(7)	0.718(8)	0.714(8)
1.5 × 1.5	0.768(10)	0.763(6)	0.763(7)	0.763(7)	0.765(6)	0.764(7)	0.763(7)	0.762(7)
1.5 × 2.0	0.784(17)	0.783(6)	0.782(7)	0.783(6)	0.784(6)	0.783(6)	0.786(6)	0.777(7)
1.5 × 3.0	0.798(5)	0.808(6)	0.801(6)	0.808(6)	0.802(6)	0.802(6)	0.809(6)	0.791(7)
2.0 × 0.5	0.555(7)	0.701(8)	0.656(10)	0.702(8)	0.639(9)	0.648(8)	0.623(8)	0.609(8)
2.0 × 0.8	0.685(4)	0.736(7)	0.719(9)	0.737(7)	0.714(8)	0.716(8)	0.701(8)	0.693(8)
2.0 × 1.0	0.725(9)	0.754(6)	0.745(8)	0.754(7)	0.743(7)	0.744(7)	0.734(7)	0.728(7)
2.0 × 1.5	0.781(5)	0.783(6)	0.781(7)	0.783(6)	0.783(6)	0.783(6)	0.778(7)	0.776(7)
2.0 × 2.0	0.800(6)	0.801(6)	0.801(6)	0.801(6)	0.803(6)	0.803(6)	0.801(6)	0.800(6)
2.0 × 3.0	0.821(15)	0.824(5)	0.822(6)	0.825(6)	0.824(5)	0.824(6)	0.827(6)	0.817(6)
3.0 × 0.5	0.567(8)	0.741(7)	0.677(9)	0.743(7)	0.654(9)	0.667(8)	0.641(8)	0.619(8)
3.0 × 0.8	0.694(12)	0.769(6)	0.738(8)	0.770(7)	0.731(8)	0.734(7)	0.718(8)	0.706(8)
3.0 × 1.0	0.736(5)	0.784(6)	0.764(7)	0.784(6)	0.761(7)	0.762(7)	0.749(7)	0.741(7)
3.0 × 1.5	0.800(12)	0.808(6)	0.801(6)	0.808(6)	0.802(6)	0.802(6)	0.793(6)	0.790(7)
3.0 × 2.0	0.816(14)	0.824(5)	0.821(6)	0.825(6)	0.824(5)	0.824(6)	0.818(6)	0.816(6)
3.0 × 3.0	0.843(8)	0.846(5)	0.846(6)	0.846(5)	0.848(5)	0.848(6)	0.846(6)	0.845(6)
4.0 × 4.0	0.878(7)	0.878(6)	0.878(6)	0.878(6)	0.880(5)	0.881(6)	0.878(6)	0.877(6)
5.0 × 5.0	0.905(9)	0.904(6)	0.904(6)	0.904(6)	0.907(6)	0.906(6)	0.904(7)	0.903(7)
10.0 × 10.0	1.000(7)	1.000(0)	1.000(0)	1.000(0)	1.003(1)	1.003(0)	1.000(0)	1.000(0)

*Note*: The uncertainties (*k* = 1) are expressed in concise notation.

As shown in Table 1, when the nominal field size was 10 cm × 10 cm, the equivalent field size closely approximated S=10cm for all models except the Sterling‐Partial Superellipse and Sterling‐Superellipse models. For these models, the equivalent field sizes were $S_{sp}=10.208\left(8\right)$ cm and $S_{ss}=10.189\left(8\right)$ cm, respectively. Consequently, for the nominal field size 10 cm × 10 cm, $\Omega {\left(S_{sp}\right)}^{fit}=\Omega {\left(S_{ss}\right)}^{fit}=1.003$, while all other models yielded $\Omega {\left(S\right)}^{fit}=1$, as indicated in Table 2. Thus, using the nominal field size 10 cm × 10 cm as reference, instead of S=10cm, would introduce a systematic 0.3% error for the Sterling‐Partial Superellipse and Sterling‐Superellipse models. To avoid redundancy, Table 2 presents $\Omega {\left(S\right)}^{exp}$ values exclusively for $S_{clin}$. For any other equivalent square small field size definition, $\Omega {\left(S\right)}^{exp}$ values can be determined by multiplying $\Omega {\left(S_{clin}\right)}^{exp}$ by the corresponding $\Omega {\left(S\right)}^{fit}$ value for the nominal 10 cm × 10 cm field.

**FIGURE 1:**
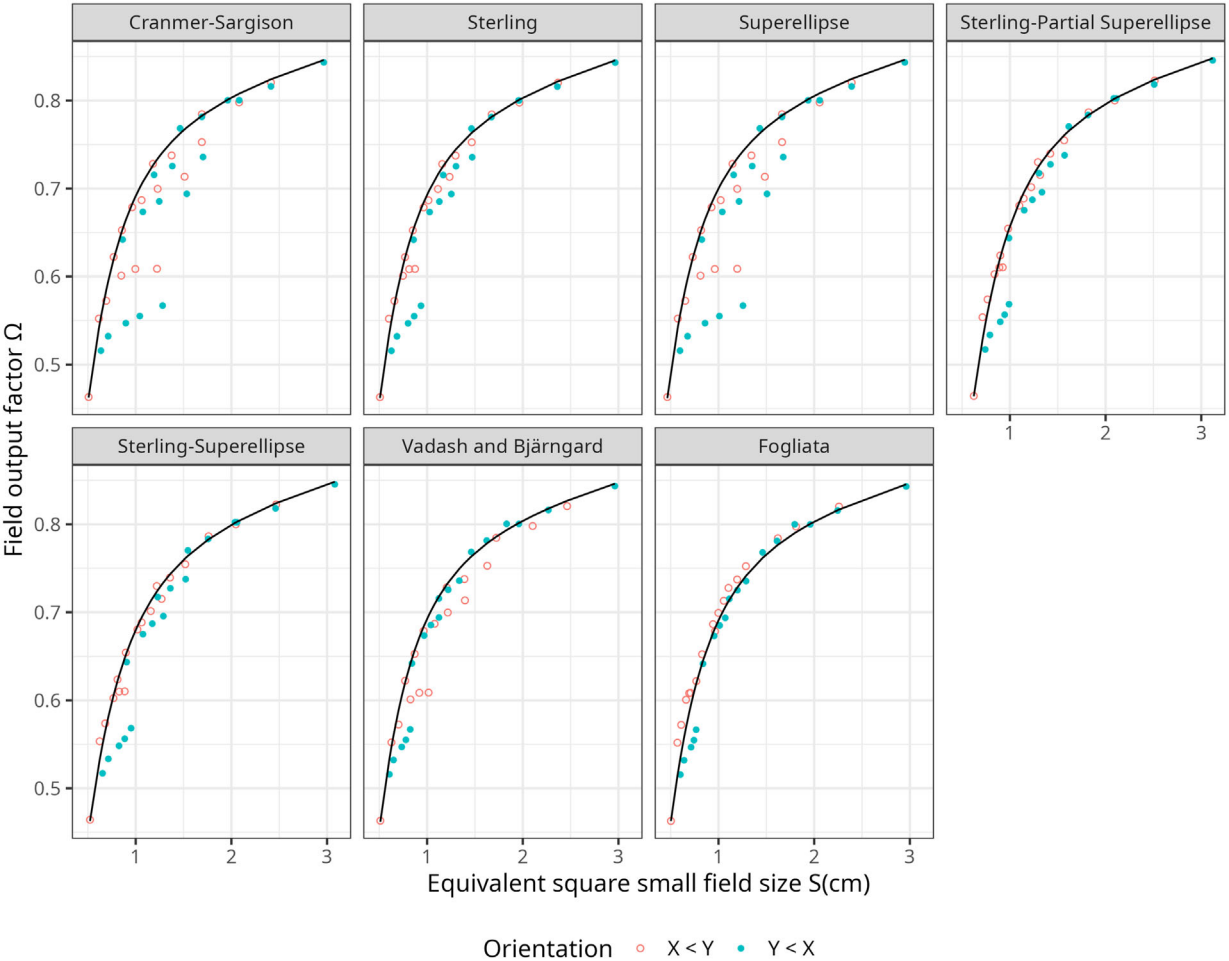
Analytical (black line) and measured (dots) field output factors are compared as a function of equivalent square field size for all models under evaluation. The collimator‐exchange effect is apparent with respect to the orientation of the field. For visual clarity, uncertainties are omitted; they are listed in Table 2.

A clearer picture of the difference between analytical and experimental FOFs for each definition of equivalent field size is displayed in Figure 1. The orientation of the field sizes highlights the collimator‐exchange effect.

### 3.2 Model comparison

Due to the stochastic component in the process of calculating $\Omega {\left(S\right)}^{fit}$ values, as well as in the parametric bootstrap resampling of uncertainties, the procedure was repeated 10 times to determine the relative log‐likelihood for each definition of equivalent square small field size. Mean and standard deviation of L for the definitions under investigation are presented in Table 3. To prevent possible outliers from affecting the results, the median and median absolute deviation (MAD) are also presented as robust estimators of the mean and standard deviation. MAD values were scaled by a factor of 1.4826 to ensure normal consistency with the standard deviation.

**TABLE 3:** Mean, standard deviation, median, and median absolute deviation (MAD) of the relative log‐likelihood of each definition of equivalent square small field size under comparison.

Definition	Mean L	St. dev.L	Median L	MAD L
Cranmer‐Sargison	−290	18	−286	19
Sterling	23	7	24	6
Superellipse	−305	13	−309	12
Sterling‐Partial Superellipse	74	4	73	6
Sterling‐Superellipse	45	5	47	7
Vadash and Bjärngard	42	9	39	8
Fogliata	113	1	113	1

The formula recently introduced by Fogliata showed the smallest discrepancy between measured and analytical values of FOFs for small rectangular fields. The second‐best results were yielded by the Sterling‐Partial Superellipse model. The Sterling‐Superellipse and Vadash and Bjärngard definitions followed. Next was the Sterling model, and lastly, the Cranmer‐Sargison and Superellipse models. With the exception of the comparisons between the Sterling‐Superellipse and Vadash and Bjärngard models, and between the Superellipse and Cranmer‐Sargison models, all differences in relative log‐likelihood were statistically significant (p<0.01) among the definitions of equivalent square field size.

## 4 DISCUSSION

**FIGURE 2:**
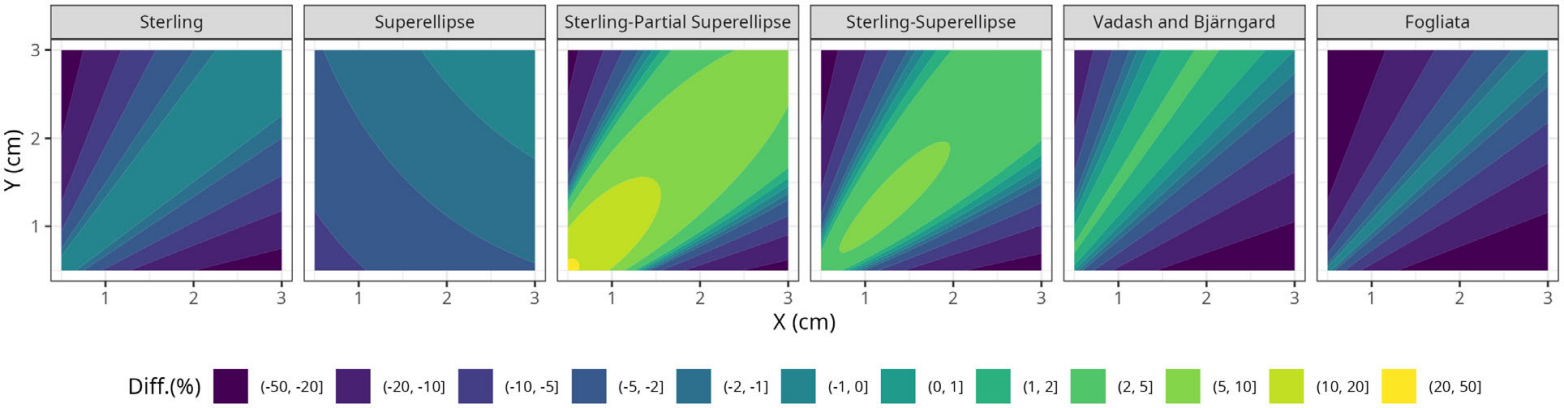
Relative differences in the equivalent square small field sizes calculated by each model compared to Cranmer‐Sargison, as a function of the *X* and *Y* dimensions of the field size.

Figure 2 presents relative differences in the equivalent square small field sizes calculated by each model compared to Cranmer‐Sargison as a function of the *X* and *Y* dimensions of the field size. For better visualization, the degree n of the superellipse was interpolated with a second‐order polynomial in both dimensions.

In this figure, it can be observed, for instance, the mitigation of the collimator‐exchange effect by the Fogliata and Vadash and Bjärngard definitions, as well as the reduction of S relative to $S_{clin}$ when the aspect ratio of the field increases for all models except the Superellipse model. Additionally, the Fogliata, Sterling, and the Vadash and Bjärngard models produce the same results for square fields as the Cranmer‐Sargison model. However, this is not the case for the definitions based on superellipses, due to their dependence on the shape of the field described by the degree n of the superellipse.

The definition of Fogliata was found to be the most accurate among the equivalent square small field size formulas evaluated in this comparison. Consequently, it shall be considered the preferred choice when the goal is to minimize uncertainties in the calculation of FOFs. However, recommending this or any alternative to the Cranmer‐Sargison definition also requires consideration of the methodological implications involved in determining FOF values using each model. In this regard, the Fogliata and Vadash and Bjärngard models include one fitting parameter that must be derived from a comprehensive set of rectangular field measurements. Interestingly, the optimal value of the fitting parameter for the Fogliata formula obtained in this work coincides with the value determined by Fogliata et al.^11^ (i.e., a=1.12). In both studies, small fields were irradiated with Varian TrueBeam linear accelerators. However, Fogliata et al.^11^ shaped the fields by using the MLC, while the present study used the jaws. To enhance the applicability of the Fogliata model, future studies should extend its evaluation to a broader range of linear accelerators, including those from different manufacturers and models. Determining the optimal value of the empirical parameter (a) for various systems would eliminate the need for individual users to perform their own fitting, thereby simplifying implementation. The Sterling‐Partial Superellipse and Sterling‐Superellipse definitions necessitate measuring the degree n of the superellipse, deviate from Cranmer‐Sargison for square fields, and involve substantially more complex calculations. The Sterling formula does not provide results as accurate as the previously mentioned models. However, it produces more accurate results than the Cranmer‐Sargison definition for rectangular fields, while converging with it for square fields and retaining computational simplicity. Finally, the Superellipse model offers the benefit of a unified formulation for square and circular fields but did not improve the accuracy of the Cranmer‐Sargison formula for small rectangular fields.

The impact on clinical dosimetry of using the Fogliata definition of equivalent square field size instead of the Cranmer‐Sargison model is clearly demonstrated in Figure 1 and highlights the limitations of the CoP for rectangular fields within 0.7<X/Y<1.4. In our study, for example, the dose difference between the experimental value and the value derived using the Fogliata definition for a nominal 0.5 cm × 3.0 cm field was –2.5%, whereas it reached 20% when using the Cranmer‐Sargison model. Such discrepancies and limitations should be considered when comparing vendor‐supplied data with user‐measured data for small rectangular fields during treatment planning system commissioning.

## 5 CONCLUSION

In the IAEA TRS‐483 CoP, rectangular small field sizes are approximated to equivalent square small fields by using the Cranmer‐Sargison definition. The purpose of the present investigation was to compare the accuracy of this definition against other formulas of equivalent square small fields. The accuracy of Cranmer‐Sargison, Sterling, Superellipse, Sterling‐Partial Superellipse, Sterling‐Superellipse, Vadash and Bjärngard, and Fogliata definitions was compared by identifying the most accurate definition of equivalent square field size as the one yielding the smallest discrepancy between measured and analytical FOFs values for rectangular fields. The log‐likelihood of the measurements was selected as metric. The Fogliata model produced the most accurate results, followed by Sterling‐Partial Superellipse, Sterling‐Superellipse, Vadash and Bjärngard, Sterling, Cranmer‐Sargison, and lastly, the Superellipse model. The differences in relative log‐likelihood were statistically significant (p<0.01) between all definitions of equivalent square field size except between the Sterling‐Superellipse and Vadash and Bjärngard models and between the Superellipse and Cranmer‐Sargison models.

These findings suggest a potential improvement to the IAEA TRS‐483 CoP, enhancing the accuracy of field output correction factors for rectangular small fields.

## AUTHOR CONTRIBUTIONS

**Ignasi Méndez**: Conceptualization; methodology; data analysis; writing, and editing. **Mateb Al Khalifa**: Data collection; manuscript review, and editing. **Haya Aljuaid**: Data collection; manuscript review, and editing. **Božidar Casar**: Conceptualization; methodology; funding acquisition; manuscript review, and editing.

## CONFLICT OF INTEREST STATEMENT

The authors declare no conflicts of interest.
